# Immortalized Canine Dystrophic Myoblast Cell Lines for Development of Peptide-Conjugated Splice-Switching Oligonucleotides

**DOI:** 10.1089/nat.2020.0907

**Published:** 2021-03-25

**Authors:** Yuichiro Tone, Kamel Mamchaoui, Maria K. Tsoumpra, Yasumasa Hashimoto, Reiko Terada, Rika Maruyama, Michael J. Gait, Andrey A. Arzumanov, Graham McClorey, Michihiro Imamura, Shin'ichi Takeda, Toshifumi Yokota, Matthew J.A. Wood, Vincent Mouly, Yoshitsugu Aoki

**Affiliations:** ^1^ Department of Molecular Therapy, National Institute of Neuroscience, National Center of Neurology and Psychiatry, Kodaira, Japan.; ^2^ Discovery Research Laboratories in Tsukuba, Nippon Shinyaku Co., Ltd., Tsukuba, Japan.; ^3^ Center of Research in Myology, Sorbonne University, INSERM, Institute of Myology, Paris, France.; ^4^ Department of Medical Genetics, Faculty of Medicine and Dentistry, University of Alberta, Edmonton, Canada.; ^5^ Medical Research Council, Laboratory of Molecular Biology, Cambridge, United Kingdom.; ^6^ Department of Paediatrics, University of Oxford, Oxford, United Kingdom.; ^7^ Oxford Harrington Rare Disease Centre, University of Oxford, John Radcliffe Hospital, Oxford, United Kingdom.

**Keywords:** Duchenne muscular dystrophy, canine X-linked muscular dystrophy in Japan (CXMD_J_), immortalized dystrophic canine myoblast, splice-switching oligonucleotides, phosphorodiamidate morpholino oligomer, cell-penetrating peptide

## Abstract

Duchenne muscular dystrophy (DMD) is a severe muscle-wasting disease caused by frameshift or nonsense mutations in the *DMD* gene, resulting in the loss of dystrophin from muscle membranes. Exon skipping using splice-switching oligonucleotides (SSOs) restores the reading frame of *DMD* pre-mRNA by generating internally truncated but functional dystrophin protein. To potentiate effective tissue-specific targeting by functional SSOs, it is essential to perform accelerated and reliable *in vitro* screening-based assessment of novel oligonucleotides and drug delivery technologies, such as cell-penetrating peptides, before their *in vivo* pharmacokinetic and toxicity evaluation. We have established novel canine immortalized myoblast lines by transducing murine cyclin-dependent kinase-4 and human telomerase reverse transcriptase genes into myoblasts isolated from beagle-based wild-type or canine X-linked muscular dystrophy in Japan (CXMD_J_) dogs. These myoblast lines exhibited improved myogenic differentiation and increased proliferation rates compared with passage-15 primary parental myoblasts, and their potential to differentiate into myotubes was maintained in later passages. Using these dystrophin-deficient immortalized myoblast lines, we demonstrate that a novel cell-penetrating peptide (Pip8b2)-conjugated SSO markedly improved multiexon skipping activity compared with the respective naked phosphorodiamidate morpholino oligomers. *In vitro* screening using immortalized canine cell lines will provide a basis for further pharmacological studies on drug delivery tools.

## Introduction

Duchenne muscular dystrophy (DMD) is an X chromosome-linked, progressive, fatal degenerative muscle disorder with an estimated prevalence of 1 in 3,500–5,000 live human male births worldwide [1,2]. DMD is mainly caused by out-of-frame mutations in the *DMD* gene that encodes dystrophin, a protein indispensable for the maintenance of sarcolemmal integrity [3,4]. Currently, corticosteroid treatment is recommended as the standard therapeutic option for patients with DMD aged older than 2 years, although adverse effects such as osteopenia, weight gain, behavioral changes, and growth retardation have raised concerns over the long-term use of these drugs.

Exon skipping is a novel therapeutic approach that involves the implementation of short, synthetic splice-switching oligonucleotides (SSOs) to effectively target individual *DMD* gene mutations and restore the *DMD* reading frame [5,6]. So far, three antisense phosphorodiamidate morpholino oligomers (PMOs) have been approved by the U.S. Food and Drug Administration (FDA). The first two of these are ExonDys51^®^ (eteplirsen) and Vyondys53^®^ (golodirsen), which target the pool of DMD patients with confirmed gene mutations amenable to exon 51 or exon 53 skipping, respectively; both of these PMOs are marketed by Sarepta Therapeutics [7,8]. Recently, VILTEPSO^®^ (viltolarsen), an antisense oligonucleotide drug codeveloped by the National Center of Neurology and Psychiatry (NCNP) in collaboration with Nippon Shinyaku Co., Ltd., which targets DMD patients with confirmed mutations amenable to exon 53 skipping, has received conditional approval in both Japan and the United States, and is awaiting the results of phase 3 trial (NCT04060199) to confirm its efficacy and clinical benefit [9–11].

In addition, SSOs targeting other exons of the *DMD* gene are also under development. However, the use of PMOs in animal studies is limited by their poor cellular uptake and inability to successfully target *DMD* in the diaphragm and heart. To overcome these limitations, SSOs have been conjugated to short cell-penetrating peptides, generating the so-called peptide-conjugated PMOs (PPMOs), which are the most promising candidates awaiting clinical trials [12].

Canine X-linked muscular dystrophy in Japan (CXMD_J_), a beagle-based dystrophic dog, is a midsize mammalian translational model of DMD (Supplementary Fig. S1a) that mimics the human DMD phenotype more closely than the widely used dystrophin-deficient rodent models [13] and has been used to investigate molecular mechanisms triggering the physiopathological modifications in DMD [14–19]. The CXMD_J_ dog model harbors a splice site mutation in intron 6 of the *DMD* gene on the X chromosome, which causes the loss of exon 7 and a disrupted reading frame in *DMD* mRNA (Supplementary Fig. S1b) [20]. Since restoring the *DMD* mRNA reading frame in CXMD_J_ requires multiexon skipping of exons 6 to 8, CXMD_J_ is a suitable model for conducting preclinical *in vivo* pharmacokinetic and toxicological assessments to validate the efficacy of multiexon skipping therapeutic strategies targeting a broader range of patients with DMD [21–23].

To reduce the number of animals used in the SSO drug screening process, for both ethical reasons and cost-effectiveness, reliable dystrophic cellular models exhibiting the fundamental features of DMD need to be established for effective high-throughput screening before *in vivo* validation [24,25]. Although primary myoblasts of DMD animal models have been successfully isolated and cultured, the efficacy and repeatability of such *in vitro* screening assays have been hindered by the limited proliferative capacity of the cells [26–28]. Furthermore, primary myoblasts often progressively lose myogenicity during passages in cell culture and are prone to senescence, rendering the application of dystrophin restoration strategies challenging. Moreover, the ability of myoblasts to fuse into myotubes varies owing to the heterogeneity of the cell populations harvested from individual muscle biopsies [27,29], compromising the reproducibility and validity of the assay *per se.*

To overcome these challenges and efficiently validate our SSO cocktails, we aimed to create immortalized wild-type (WT) and dystrophic canine myoblast cell lines with enhanced proliferative capacity. These myoblast cell lines can be used as valuable and cost-effective SSO screening tools before injecting the most promising SSO combination into CXMD_J_ dog for assessing its pharmacokinetics and efficacy. To this end, we immortalized canine myoblasts by transducing myoblasts isolated from 2-month-old WT or CXMD_J_ dogs with murine cyclin-dependent kinase 4 (*Cdk4*) and human telomerase reverse transcriptase (*hTERT*). In this approach, *hTERT* and *Cdk4* were, respectively, used for telomere elongation and inhibition of the stress pathway mediated by p16^INK4a^, an inhibitor of the CDK4 family [30,31]. The combination of these two genes has been shown to preserve the characteristics of the original myoblasts through immortalization [32]. To assess the extent of applicability of these immortalized myoblast lines as a suitable tool for the evaluation of therapeutic agents for DMD, the efficacy of combinations for multiexon skipping was evaluated. In addition, the drug delivery potential of a newly designed PMO-conjugated internalization peptide (Pip), called Pip8b2–PMO [33], was compared with the equivalent naked PMO cocktail. In this study, we have successfully generated dystrophic deficient immortalized cell lines that can retain their myogenic potential even after extensive passaging, and used these new tools to demonstrate efficient drug-related dose/response exon skipping and dystrophin restoration in the presence of canine PMO or PPMO SSO cocktails.

## Materials and Methods

### Ethics approval

WT and CXMD_J_ dogs were maintained at the laboratory animal facilities of the National Institute of Neuroscience, NCNP, Japan, following guidelines of the Ethics Committee for the Treatment of Laboratory Middle-Sized Animals (approved no. 2019040). Animal housing followed the basic policies for the conduct of animal experimentation outlined by the Ministry of Health, Labor and Welfare of the Center for Accreditation of Laboratory Animal Care and Use, Japan Health Sciences Foundation (Certification number: A17–130). *In vitro* cell-based assays were performed using primary and immortalized canine myoblasts and were approved by the Institutional Animal Experiment Committees of the NCNP.

### Cell immortalization

Myoblasts derived from two CXMD_J_ dogs and one WT dog (Supplementary Table S1) were immortalized as previously described [34]. Briefly, lentiviral vectors encoding *hTERT* and *Cdk4* with puromycin and neomycin selection markers, respectively, were used to transduce primary cultures of myoblasts. The transduced cells were then selected with puromycin (0.2 μg/mL) or neomycin (0.3 mg/mL) for 8 days. The transduced cells were purified as described previously [34] if necessary, and then seeded at clonal density. Individual myogenic clones expressing desmin (*DES*) were isolated from each population using glass cylinders.

### Cell proliferation assay

Cells were seeded into 96-well plates with growth medium containing Dulbecco's modified Eagle's medium/F-10 1:1 (Invitrogen, San Diego, CA), 20% fetal bovine serum, basic fibroblast growth factor (2.5 ng/mL), and 1% penicillin/streptomycin. One and 3 days later, the number of viable cells was determined using the Cell Counting Kit-8 (CCK-8; Dojindo Laboratories, Kumamoto, Japan). Absorbance at 450 nm was measured using the Synergy HTX Multi-Mode Reader (BioTek Instruments, Winooski, VT). The isolated individual clones were expanded, representing over 20 divisions from the time of transduction.

### *In vitro* cell transfections

We designed the following two antisense PMO sequences to target each of exons 6 and 8 of dog *DMD* mRNA, as previously reported [23,35]: dog_Ex6A, 5′-GTTGATTGTCGGACCCAGCTCAGG-3′; dog_Ex6B, 5′-ACCTATGACTGTGGATGAGAGCGTT-3′; dog_Ex8A, 5′-CTTCCTGGATGGCTTCAATGCTCAC-3′; and dog_C8A, 5′-GATAAGTGGTGGCAACATCTGT-3′; Standard control PMO, 5′-CCTCTTACCTCAGTTACAATTTATA-3′; dog_C8A (invert antisense), 5′-TGTCTACAACGGTGGTGAATAG-3′. PMO SSOs were purchased from Gene Tools (Philomath, OR) and were either used as naked PMOs or were conjugated with Pip8b2 [33] at the University of Oxford.

Immortalized myoblasts (5 × 10^4^ cells for 24-well plate, 1.9 × 10^4^ cells for 48-well plate) from the CXMD_J_ dogs were cultured in growth medium containing Dulbecco's modified Eagle's medium/F-10 1:1 (Invitrogen), 20% fetal bovine serum, basic fibroblast growth factor (2.5 ng/mL), and 1% penicillin/streptomycin for 24 h, then changed to a differentiation medium containing Dulbecco's minimum essential medium with 2% horse serum, and cultured for 3 days. The medium was then replaced with the same differentiation medium containing a final concentration of selected PMOs. The dual Ex6A and C8A PMO cocktail was evaluated by adding 5, 10, or 20 μM (2.5, 5, or 10 μM for each PMO) antisense Ex6A and C8A PMOs with Endo-Porter transfection reagent (Gene Tools). The efficacy of dystrophin restoration of the Ex6A, Ex6B, and Ex8A PMO cocktail and that of the PPMO was compared by adding 0.9–4.5 μM PMO compounds (0.3–1.5 μM for each PMO) and 0.3–0.9 μM PPMO compounds (0.1–0.3 μM for each PPMO) in total, respectively, without Endo-Porter. In both cases, cells were cultured for three additional days before RNA analyses or for 6 to 7 days before protein expression analyses via western blotting or immunocytochemistry.

### Reverse transcription polymerase chain reaction and quantitative polymerase chain reaction

The expressions of myogenic regulatory factors (MRFs) and markers in immortalized cells were analyzed using reverse transcription polymerase chain reaction (RT-PCR). Briefly, 500 ng of total RNA was extracted from each cell line with the RNeasy Mini kit (Qiagen, Hilden, Germany), and used for cDNA synthesis using the High Capacity cDNA synthesis kit and random hexamer primers (Thermo Fisher Scientific, Waltham, MA, USA). The obtained cDNA (3 μL) was then used as a template for PCR with the Ex Taq Hot Start Version kit (Takara Bio, Shiga, Japan) to amplify myogenic differentiation 1 (*MYOD1*), myogenin (*MYOG*), neural cell adhesion molecule 1 (*NCAM1*)*,* and *DES* with specific primers designed using the Primer3plus online tool (Supplementary Table S2). The PCR program was as follows: heat denaturation at 95°C for 5 min; 35 cycles of denaturation at 94°C for 30 s, annealing at 60°C for 30 s, and extension at 72°C for 30 s; followed by a final extension at 72°C for 10 min. PCR products were analyzed with MultiNA (Shimadzu, Kyoto, Japan).

Exon skipping was verified by PCR amplification of cDNA templates with 0.6 mM of primer spanning exons 3–4 (GGCAAAAACTGCCAAAAGAA) and exon 10 (TGCTTCGGTCTCTGTCAATG) of the canine *DMD* transcript, followed by MultiNA analysis. Exon-skipping efficiency was calculated as follows: (exon-skipped transcript molarity)/(native + intermediate + exon-skipped transcript molarity) × 100%. To ensure the identity of the exon skipped band, the amplified PCR region corresponding to the 221 bp in-frame exon skipped band was electrophoresed on agarose gels and then excised from the gels, purified using a gel extraction kit (Qiagen), and subjected to Sanger sequencing, which was performed by Eurofins Genomics (Tokyo, Japan) using the same primer set that was used for quantitative PCR (qPCR). PowerUP SYBR Green Master Mix (Thermo Fisher Scientific) was used to quantify cDNAs using StepOnePlus Real-Time PCR Systems (Thermo Fisher Scientific). Relative quantity of the genes was calculated by the ΔΔC_T_ method. Hypoxanthine phosphoribosyltransferase 1 (*HPRT 1*) and succinate dehydrogenase complex, subunit A (*SDHA*) were used as multiple reference genes. Data were analyzed using StepOne Software v2.3 (Thermo Fisher Scientific).

### Immunocytochemistry

Cells were washed with phosphate-buffered saline (PBS), fixed in 100% methanol for 10 min at −20°C, and permeabilized in 0.1% Triton-X (FUJIFILM Wako Pure Chemical, Osaka, Japan) for 10 min at room temperature. Cells were blocked with 5% goat serum for 15 min at 37°C and then incubated overnight at 4°C with mouse anti-dystrophin (1:150 dilution, NCL-Dys1; Leica Microsystems, Tokyo, Japan) and mouse anti-myosin heavy chain (MyHC) (1:400 dilution, MAB4470, clone MF20; R&D, Minneapolis, MN) antibodies. Cells were then washed with PBS and incubated with Alexa Fluor 488 goat anti-mouse IgG (H+L; 1:500 dilution, Thermo Fisher Scientific) as the secondary antibody for 60 min at room temperature. Nuclei were stained with 4,6-diamidino-2-phenylindole (DAPI) solution (1:1,000 dilution, DOJINDO). Labeling signals were imaged using a fluorescent microscope (BZ-X800, KEYENCE, Osaka, Japan) and BZ-X Analyzer or BZ-X800 Analyzer (KEYENCE).

### Western blotting

Sample preparation, sodium dodecyl sulfate/polyacrylamide gel electrophoresis (SDS-PAGE), and semidry blotting were performed as previously described [36]. After blocking for 1 h in 5% skimmed milk, polyvinylidene fluoride (PVDF) membranes containing the proteins were incubated with the following primary antibodies: mouse anti-dystrophin (1:500 dilution, ab15277; Abcam, Cambridge, United Kingdom or 1:500 dilution, NCL-Dys1; Leica Microsystems) and mouse anti-alpha-actinin (1:2,000 dilution, A7811, Sigma-Aldrich) as a loading control. This was followed by incubation with the secondary antibody Histofine Simple Stain MAX PO (MULTI) (1:500 dilution; Nichirei Biosciences, Tokyo, Japan). The bands were visualized using SuperSignal West Dura Extended Duration Substrate (Thermo Fisher Scientific) in ChemiDoc Touch MP Imaging System (Bio-Rad Laboratories, Hercules, CA) and quantified using ImageLab software (Thermo Fisher Scientific).

### Statistical analysis

Statistical analysis and calculation of effective concentration (EC_50_) were conducted using GraphPad Prism 8 (GraphPad Software, La Jolla, CA). Student's t-test or one-way analysis of variance with parametric Dunnett's multiple comparisons test was conducted to determine statistical significance. *P* < 0.05 was considered statistically significant.

## Results

### Successful generation of immortalized canine WT and dystrophin-deficient myoblast lines

**FIG. 1.**
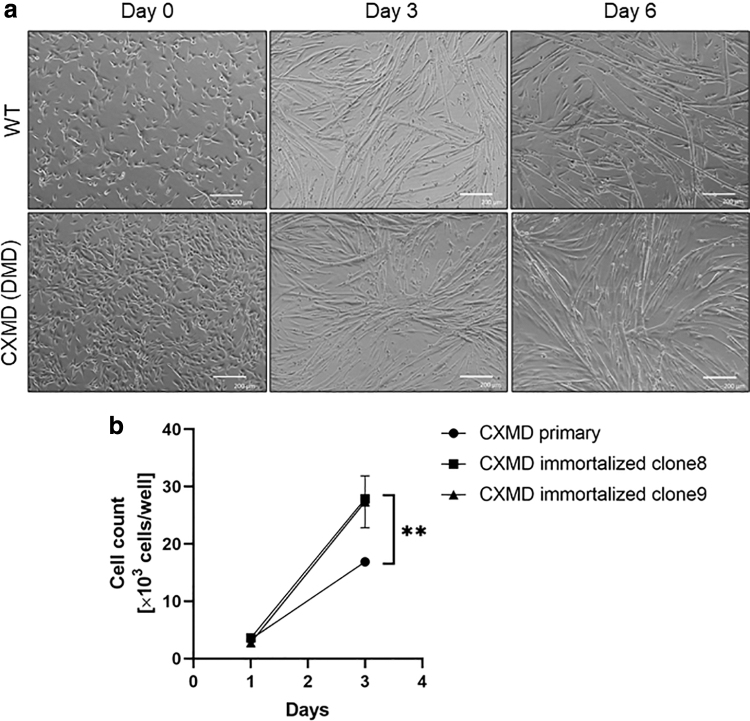
Differentiation and proliferation ability of immortalized WT and dystrophic canine myoblasts. **(a)** Representative phase-contrast images of cells undergoing differentiation. Images of immortalized WT clone 2 and CXMD_J_ clone 9 were obtained after 0, 3, or 6 days of differentiation. Scale bar: 200 μm. **(b)** Cell proliferation assay using Cell Counting Kit-8. Cell counts per well are represented as the mean ± standard deviation. *N* = 3; **P < 0.01. CXMD_J_, canine X-linked muscular dystrophy in Japan; WT, wild type.

**FIG. 2.**
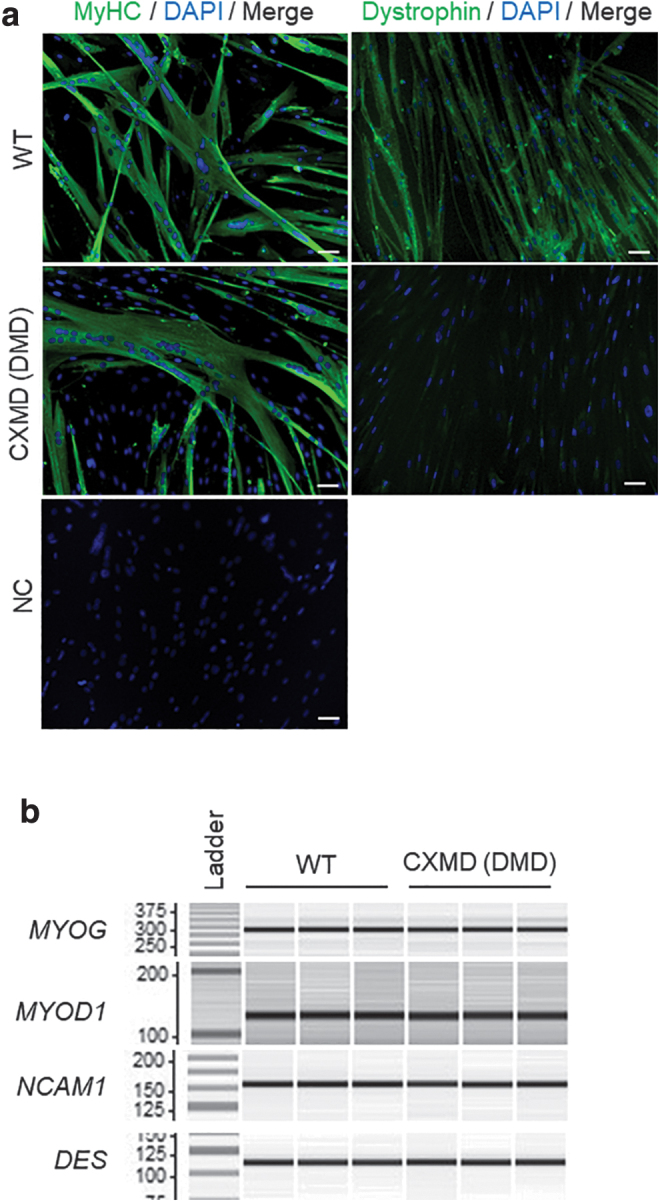
Myogenic properties of newly established cell lines from WT and dystrophic dogs. **(a)** Immunocytochemistry showing MyHC expression on day 6 and dystrophin on day 8 after triggering differentiation in WT clone 2 and CXMD_J_ clone 9 (*green*), respectively. Nuclei were visualized with DAPI (*blue*). Scale bar: 50 μm. NC, negative control without primary antibody. **(b)** Expression of muscle regulatory factors, including *MYOD1*, *MYOG*, *NCAM1*, and *DES*, evaluated by RT-PCR of immortalized WT clone 2 and CXMD_J_ clone 9. *N* = 3. *DES*, desmin; MyHC, myosin heavy chain; *MYOD1*, myogenic differentiation 1; *MYOG*, myogenin; *NCAM1*, neural cell adhesion molecule 1; RT-PCR, reverse transcription polymerase chain reaction.

We first tested the differentiation and proliferation capabilities of our novel immortalized myoblast cell lines. For further characterization, we chose two clones (clone 8 and 9) out of six, and two clones (clone 2 and 3) out of three for CXMD_J_ and WT immortalized myoblast cell lines, respectively, based on their higher ability to proliferate and form fully mature myotubes when subjected to differentiation (data not shown). After culturing for 6 days under differentiation conditions, all immortalized myoblasts fused into myotubes with the expression of skeletal muscle MyHCs (Figs. 1a, 2a, and Supplementary Fig. S2a, b). In addition, dystrophin expression was only detected in immortalized myoblasts from WT dogs by immunocytochemistry, as expected (Fig. 2a). The immortalized cells for at least 20 consecutive passages after transduction showed superior ability to proliferate compared with their respective parental primary myoblasts, which were passaged 15 times (Fig. 1b). Furthermore, this superior ability was maintained in all clones, indicating that the immortalization process inhibited cellular senescence.

### Expression of MRF and myogenic markers during differentiation of immortalized myoblast cell lines

To confirm that the WT and dystrophic immortalized cell lines maintained their myogenic signatures, we investigated the expression of several myogenic markers that have previously been used as key validators of myogenic status [32]. After 6 days of differentiation, WT and dystrophic-derived cell lines expressed MRFs and the myogenic markers *MYOG*, *MYOD1*, *NCAM1*, and *DES* (Fig. 2b and Supplementary Fig. S2b). The formation of myotubes in primary myoblasts from CXMD_J_ dogs (with 15 passages) was weaker than in the immortalized myoblasts (Supplementary Fig. S2d). In line with this finding, the expression of *MYOG* was undetectable by qPCR (data not shown), while the expression levels of *NCAM1* and *DES* were lower in the primary myoblasts after 15 passages compared with the immortalized myoblasts, suggesting that maintenance of the myogenic capacity of myoblasts is difficult, possibly due to the dominant proliferation of originally pertained fibroblasts in the late passages (Supplementary Fig. S2d).

### Successful skipping of exons 6/8 in immortalized dystrophic canine myoblast cell lines using a dual PMO cocktail

**FIG. 3.**
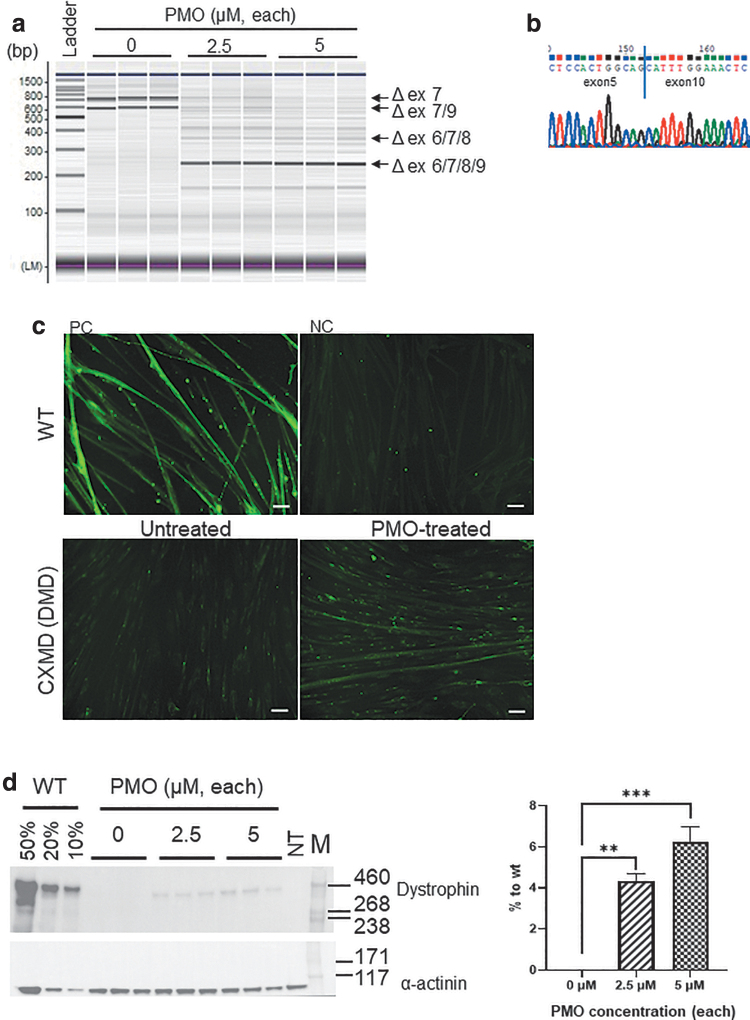
Multiexon skipping analysis using dual cocktail of PMOs targeting exons 6 and 8 of *DMD* in immortalized myoblast lines. **(a)** RT-PCR of immortalized CXMD_J_ clone 9 with or without PMO treatment. *N* = 3. **(b)** Direct sequencing of the PCR products of the skipped transcript in **(a)**. **(c)** Immunocytochemistry of dystrophin in WT clone 2 and CXMD_J_ clone 9 with or without PMO treatment. NC, negative control with absence of primary antibody against dystrophin; PC, positive control with WT clone 2. Scale bar: 50 μm. **(d)** Western blotting analysis for dystrophin expression. Protein expression levels are represented as the mean ± standard deviation, normalized to the level of alpha-actinin. *N* = 3; **P < 0.01, ***P < 0.001. NT, not treated; M, high-molecular-weight protein marker. DMD, Duchenne muscular dystrophy; PMOs, phosphorodiamidate morpholino oligomers.

CXMD_J_ dogs harbor a splice site mutation in the acceptor site of intron 6, leading to an out-of-frame mRNA transcript fusing exons 6 to 8 (Supplementary Fig. S1b). Skipping of exons 6 to 8 results in exon 6 to 8 or 6 to 9 multiexon skipping, due to endogenous alternative skipping of exon 9. When the immortalized myoblast lines from CXMD_J_ dogs were treated with a cocktail of the PMOs Ex6A and C8A, targeting exons 6 and 8 of the *DMD* gene, respectively, exon skipping products, especially exon 6 to 9 multiexon skipping, were successfully detected by RT-PCR (Fig. 3a, b). Moreover, the apparent restoration of the *DMD* mRNA reading-frame was confirmed by western blotting and immunocytochemistry (Fig. 3c, d). The restoration levels of dystrophin in dystrophic canine myoblast cell lines treated with individual PMO concentrations of 2.5 or 5 μM were around 4% and 6%, respectively. Using the immortalized myoblast line from WT dogs, we also detected exon skipping caused by the PMO cocktail chosen by us (Supplementary Fig. S3a, b).

### Successful skipping of exons 6/8 in immortalized dystrophic canine myoblast cell lines using a combination of three SSO-based PMO or PPMO cocktail

Pip8b2 is a recently optimized peptide demonstrating higher cell-penetrating efficiency than the previously reported members of the Pip series [33]. We sought to investigate this improved potency through comparison of a previously reported three SSO-based PMO cocktail comprising Ex6A, Ex6B, and Ex8A [23] versus that of the respective Pip8b2-conjugated cocktail, using our newly generated immortalized dystrophin-deficient cell line.

**FIG. 4.**
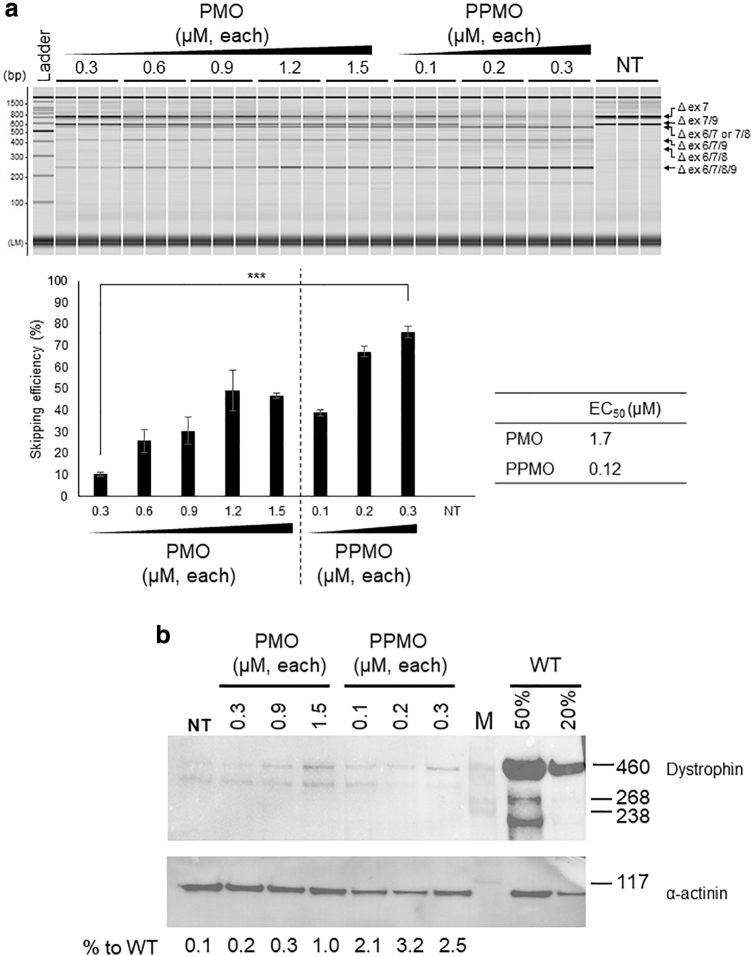
Multiexon skipping using the three-PPMO-based cocktail in immortalized myoblast lines. **(a)** RT-PCR of immortalized CXMD_J_ clone 9 treated with PMO or PPMO. Exon 6 to 9 skipping efficiency for each PMO and PPMO compound is depicted as a bar graph and EC_50_ values are noted on the *right*-hand site. Exon skipping efficiency is represented as the mean ± standard deviation. *N* = 3; ***P < 0.001. NT, not treated. **(b)** Western blotting analysis for dystrophin expression. M, high-molecular-weight protein marker. EC_50_, effective concentration; PPMO, Pip8b2-conjugated PMO.

When cells were transfected by a mixture of PMO or Pip8b2-PMO without any transfection reagent (gymnotic delivery), dose-dependent exon 6 to 9 skipping was observed. In the case of the PMO cocktail, the exon skipping efficiency seemed to be saturated at 1.2 μM, whereas the Pip8b2-conjugated PMO cocktail showed much higher skipping efficiency at just 0.3 μM. The half-maximal EC_50_ of exon skipping activity was ∼14 times lower in the cells transfected by Pip8b2-PMOs than in those transfected by the unconjugated PMO cocktail (Fig. 4a). Western blotting analysis revealed a higher expression level of dystrophin in Pip8b2-PMO-transfected cells compared with that in unconjugated PMO cocktail-transfected cells (Fig. 4b). The exon skipping efficiency observed in this assay was consistent with the same assay performed using primary myoblasts between passages 4–10 (Supplementary Fig. S4). We also confirmed that exon skipping was not induced by transfection of standard control PMO as recommended by Gene Tools and, dog_C8A (invert antisense) conjugated to Pip8b2 (refer to Materials and Methods section) (Supplementary Fig. S5).

## Discussion

Over the last decade, there has been a surge in the field of translational research aiming to develop novel gene-based therapeutic approaches for treating DMD, given the fact that current treatment regimens only alleviate the severity or delay the progression of DMD, without successfully reverting the pathophysiological phenotype or eradicating the cause of this disease. Exon skipping has emerged as a promising therapeutic approach for the treatment of patients with DMD; however, the clinical benefit of the three FDA-approved PMOs, eteplirsen, golodirsen, and viltolarsen, requires further confirmatory studies [37]. Although a plethora of potent peptide-conjugated PMO derivatives, called PPMOs, are currently being tested to enhance exon skipping treatment methods in laboratories worldwide, their transition from laboratory animal experimentation to human clinical trials will be dependent on improvement of their therapeutic profile due to inherent toxicity issues that surround the use of cationic-based peptides [12]. To effectively overcome this challenge, high-throughput screening of novel peptide-conjugated SSOs in cellular systems derived from dystrophic animal models that more closely mimic the human DMD phenotype needs to be developed.

Our laboratory is currently committed to developing SSO-based treatments targeting a wide range of the DMD patient pool. To accurately test the efficacy, tolerability, and toxicity of novel SSO agents, we are breeding various dystrophin-deficient mouse models and have routinely screened SSOs using primary and immortalized model-specific myoblasts. Given the fact that all our in-house mouse dystrophic models (*mdx*, *mdx52*, human *DMD*-null) present with a mild nonprogressive form of DMD, and are characterized by acute necrosis at an early age and rapid muscle regeneration, we opted to use the CXMD_J_ dog model as an extra confirmatory step to study the pharmacokinetics of our most promising SSO PMO and PPMO-based cocktails. In fact, CXMD_J_ is an ideal midsized canine model for proof-of-concept studies on multiexon 6 to 8 skipping, genome editing, and viral-based and cell-based therapies for DMD, which presents with a severe and progressive form of DMD, with histopathological characteristics that more closely resemble those in humans [22,23]. Nevertheless, before the assessment of such strategies *in vivo*, *in vitro* screening assays are required to limit animal experimentation as well as save time and expenses. We have previously used primary myoblasts isolated from dogs for this purpose; however, based on our experience, these cells generally show relatively less proliferation, imperfect myotube differentiation, and variable drug response in advanced passages, owing to the high heterogeneity of the cell population [26–28,38]. To overcome these limitations, canine dystrophic myoblasts need to be isolated using fluorescence-activated cell sorting [39–41]; however, this process affects cell viability [42].

In this study, we provide the first report on the successful establishment of canine immortalized myoblasts from skeletal muscles of CXMD_J_ and WT beagle dogs. An immortalized canine myoblast cell line was recently established from a normal Golden Retriever/Labrador mix dog using SV40 as an immortalizing agent [43], however, it has been shown that the expression of SV40 Tag perturbs myogenic differentiation [44]. Moreover, there has not been any report of myoblast line originating from canine dystrophic models, which is an indispensable tool for the estimation of EC_50_ and exon skipping efficiency of SSO cocktails. In general, immortalization of myoblasts is associated with a high risk of loss of myogenesis owing to prolonged expansion of cells [45–48]. However, we have previously demonstrated successful establishment of immortalized pathological human myoblasts from the myoblasts of patients with various neuromuscular disorders and confirmed that their myogenic characteristics are well-maintained [32,34]. Using the same method, we have established immortalized canine dystrophic myoblasts and confirmed that the clones selected for analysis demonstrate significantly enhanced proliferation and myotube formation abilities compared with those of the primary culture cells from which these immortalized cells originated. This differentiation was accompanied by the expression of several myogenic markers, in line with the previous report of immortalized human myoblasts [34,49]. Even in late passages, myogenicity was well-maintained in immortalized myoblasts compared with that in primary myoblasts, confirming the appropriateness of these novel immortalized cell lines to use as a screening tool for therapeutics in skeletal muscle. Thus, our dystrophic myoblast lines can be used to screen for modified SSOs and the results obtained may be useful for comparisons of *in vitro* dystrophin restoration potency between existent and already trialed compounds versus novel-synthesized compounds, allowing for estimations of minimal concentrations best utilized *in vivo* to produce the desired effect.

In addition, we evaluated the recently designed cell penetrating peptide of the Pip series, named Pip8b2, using the immortalized CXMD_J_ myoblast line. The generation of Pip8b2, which belongs to the novel series of CPPs, was based on the observation that the amino acids composing the hydrophobic cores of the peptides, rather than the linear amino-acid sequences, predominantly determine the cell penetration potency (EP2751128A2, European Patent Office). As expected, we verified that Pip8b2-conjugated PMO cocktails markedly improved the exon 6 to 9 skipping activity of corresponding naked PMOs, in line with dose/response PMO and PPMO experiments carried out using our primary myoblast cell lines. This result encourages *in vivo* studies involving pharmacological, pharmacokinetic, and toxicological analyses.

In conclusion, we assessed the improved exon skipping efficiency of PMOs due to Pip8b2 conjugation, using the newly established dystrophic myoblast line as a tool. The same assay can be applied for further optimization of PPMOs and other modified SSOs.

## Supplementary Material

Supplemental data

Supplemental data

Supplemental data

Supplemental data

Supplemental data

Supplemental data

Supplemental data
