# Supplementary material for: Immortalized Canine Dystrophic Myoblast Cell Lines for Development of Peptide-Conjugated Splice-Switching Oligonucleotides
Source: Nucleic Acid Ther. 2021 Mar 25;31(2):172–81. doi: 10.1089/nat.2020.0907 (PMC7997716; doi:10.1089/nat.2020.0907)
Supplement: Supplemental data [file Supp_Fig1.docx]

**
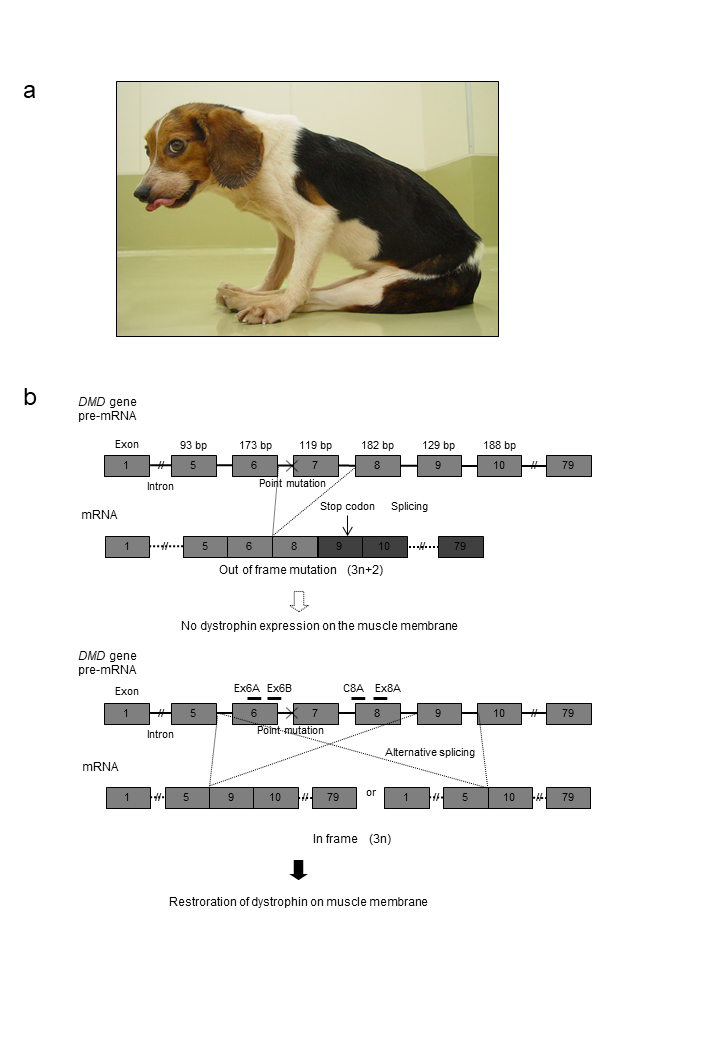
**

**Figure S1. Mutation in CXMD_J_ and strategy for exon-skipping treatment.** (a) Representative photograph of a CXMD_J_ dog. (b) Dystrophic dog harbors a point mutation at the splice site in intron 6, which leads to the lack of exon 7 in the mRNA. Single exon skipping of exon 6 or exon 8 leads to out-of-frame products. Exclusion of at least two further exons (exon 6 and exon 8) is required to restore the reading frame.
