# Supplementary material for: Immortalized Canine Dystrophic Myoblast Cell Lines for Development of Peptide-Conjugated Splice-Switching Oligonucleotides
Source: Nucleic Acid Ther. 2021 Mar 25;31(2):172–81. doi: 10.1089/nat.2020.0907 (PMC7997716; doi:10.1089/nat.2020.0907)
Supplement: Supplemental data [file Supp_Table1.docx]

**Supplementary materials**

**Table S1. Dogs used in the study to isolate myoblasts.**

|  | Dog ID |
| --- | --- |
| Wild type | 15303MN |
| CXMD_J_ | 15301MA |
| CXMD_J_ | 15302MA |

Letters in the ID indicate sex (M = male) and genotype (N = normal, A = affected, with CXMD_J_ mutation). All the dogs were sacrificed at the age of 2-months.
