# Supplementary material for: Immortalized Canine Dystrophic Myoblast Cell Lines for Development of Peptide-Conjugated Splice-Switching Oligonucleotides
Source: Nucleic Acid Ther. 2021 Mar 25;31(2):172–81. doi: 10.1089/nat.2020.0907 (PMC7997716; doi:10.1089/nat.2020.0907)
Supplement: Supplemental data [file Supp_Table2.docx]

**Table S2.** Specific PCR primers used for detection and quantification of canine myogenic regulatory factors and myogenic markers.

| Target | Sequence |
| --- | --- |
| *MYOD1* | AACACTACAGCGGCGACTC |
|  | TAGGTGCCATCGTAGCAGTTC |
| *DES* | TTGGAGCGCAGGATTGAATC |
|  | ACCTGTTGTTCCTGAAGCTG |
| *NCAM1* | CGGCCACGCTGAGGGAAAG |
|  | GGAAAGGCCGCAGAGACG |
| *MYOG* | CGGCCACGCTGAGGGAAAAG |
|  | GGAAAGGCCGCAGAGACG |
| *HPRT1* | TGCTCGAGATGTGATGAAGG |
|  | TCCCCTGTTGACTGGTCATT |
| *SDHA* | TGACTCTGGAAATCCGTGAAGG |
|  | AAATGCCAGGCAGACGTATG |
