# Supplementary material for: Immortalized Canine Dystrophic Myoblast Cell Lines for Development of Peptide-Conjugated Splice-Switching Oligonucleotides
Source: Nucleic Acid Ther. 2021 Mar 25;31(2):172–81. doi: 10.1089/nat.2020.0907 (PMC7997716; doi:10.1089/nat.2020.0907)
Supplement: Supplemental data [file Supp_Fig2.docx]

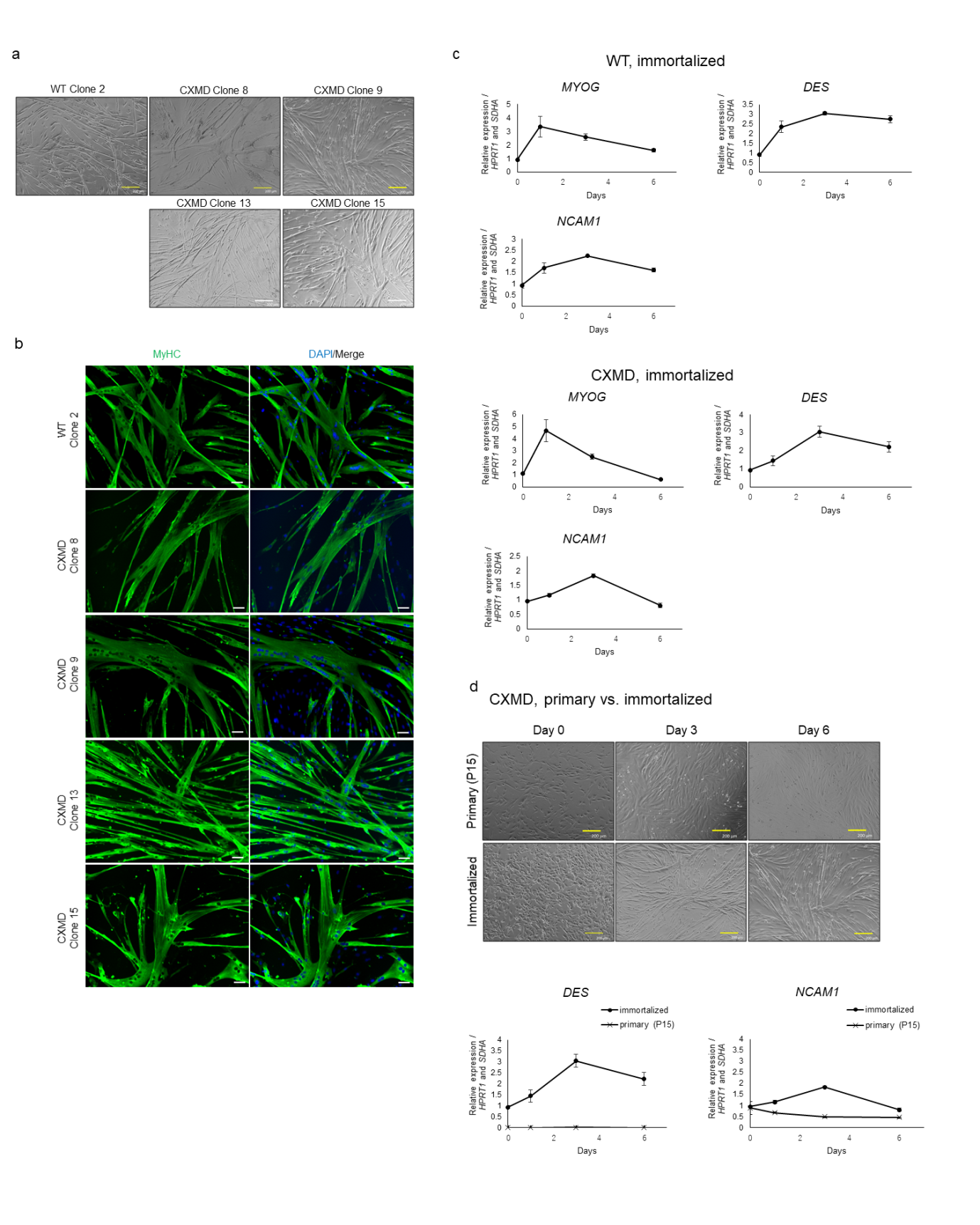


**Figure S2. Myogenic properties of the newly established cell lines from WT and dystrophic dogs.** (a) Representative phase-contrast images of cells that underwent differentiation. Images were obtained after 6 days of differentiation. Scale bar: 200 μm. (b) Immunocytochemistry of myosin heavy chain (MyHC) at day 6 after differentiation (green). Scale bar: 50 μm. (c) Time-course change of myogenic regulatory factor (MRF) expression level (qPCR, ΔΔCt, N = 3). cDNA from differentiated immortalized CXMD_J_ clone 9 and WT clone 2 were analyzed by qPCR. Each expression level is presented as the mean ± standard deviation, normalized to the level of *HPRT 1* and *SDHA* expression. (d) Comparison of myotube formation (scale bar: 200 μm) and time-course change of the MRF expression level (qPCR, ΔΔCt, N = 3) between primary myoblasts (passage number 15) and immortalized CXMD_J_ clone 9 under differentiation condition.
