# Supplementary material for: Immortalized Canine Dystrophic Myoblast Cell Lines for Development of Peptide-Conjugated Splice-Switching Oligonucleotides
Source: Nucleic Acid Ther. 2021 Mar 25;31(2):172–81. doi: 10.1089/nat.2020.0907 (PMC7997716; doi:10.1089/nat.2020.0907)
Supplement: Supplemental data [file Supp_Fig3.docx]

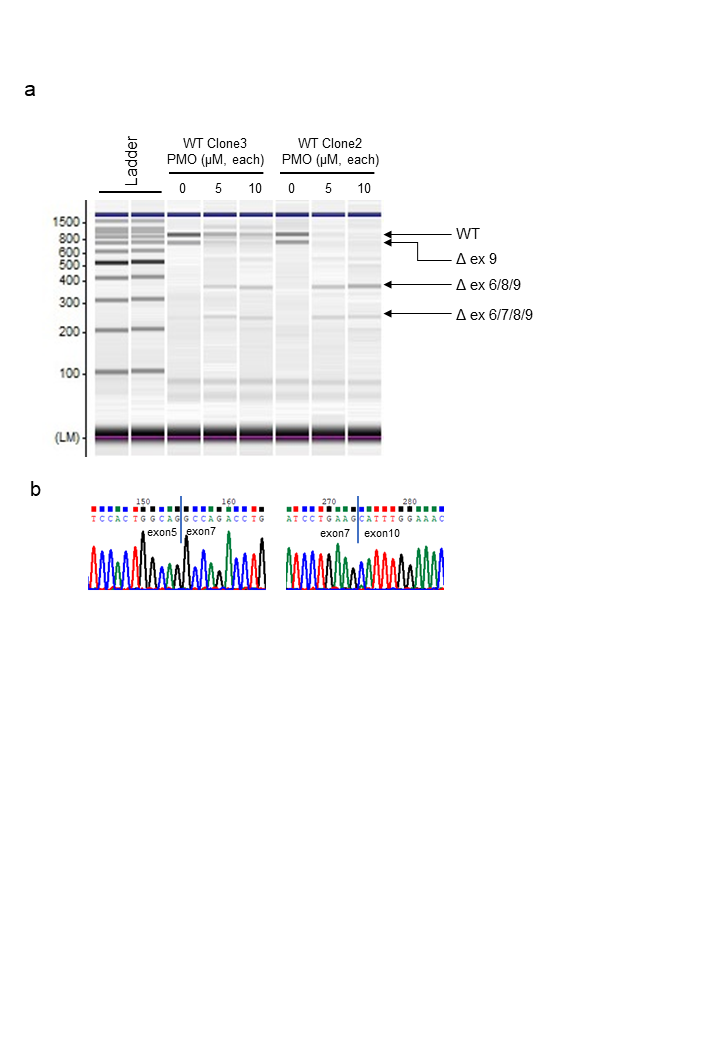


**Figure S3. Exon skipping analysis using dual cocktail of PMOs targeting exons 6 and 8 of *DMD* in immortalized myoblast lines.** (a) RT-PCR in wild type (WT) cell lines with or without PMO treatment. (b) Direct sequencing of the PCR products in (a).
