# Supplementary material for: Immortalized Canine Dystrophic Myoblast Cell Lines for Development of Peptide-Conjugated Splice-Switching Oligonucleotides
Source: Nucleic Acid Ther. 2021 Mar 25;31(2):172–81. doi: 10.1089/nat.2020.0907 (PMC7997716; doi:10.1089/nat.2020.0907)
Supplement: Supplemental data [file Supp_Fig4.docx]

**
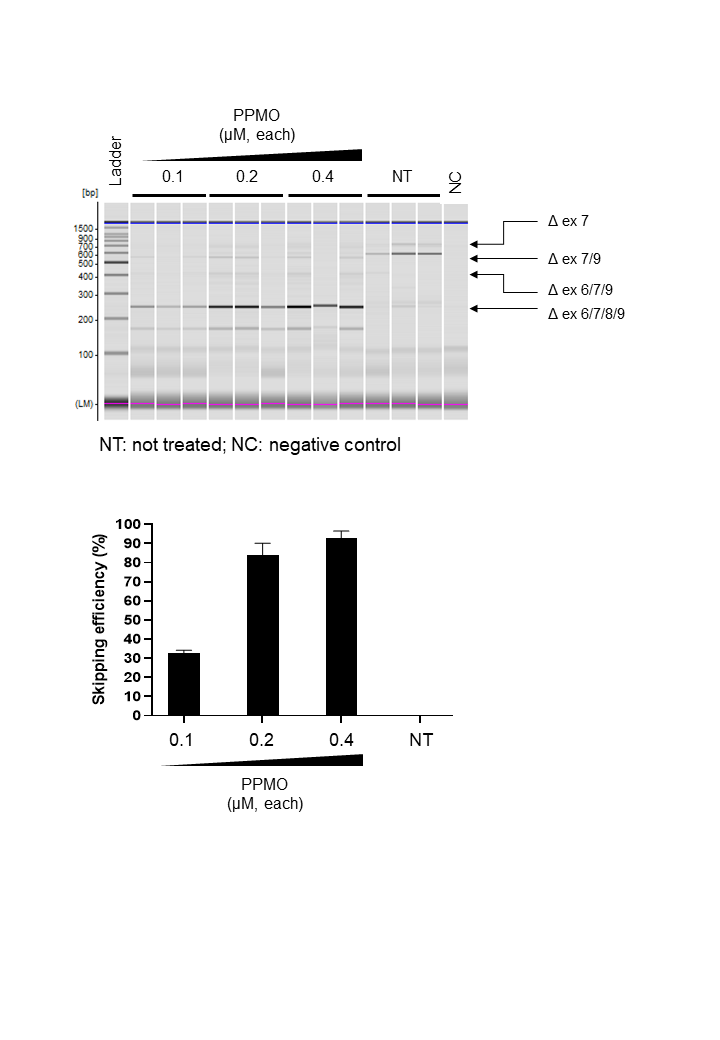
**

**Figure S4. Multi-exon skipping using the three-PPMO-based cocktail in primary CXMD_J_ myoblasts.** Representative image of RT-PCR analysis of *DMD* mRNA in primary myoblasts derived from CXMD_J_ dogs treated with three-PPMO-based cocktail, indicating successful exon 6 to 9 skipping at all concentrations used. Exon skipping efficiency are depicted as a bar graph and represented as the mean ± standard deviation. Data are representative of at least three individual experiments performed in triplicate.
