# Supplementary material for: Immortalized Canine Dystrophic Myoblast Cell Lines for Development of Peptide-Conjugated Splice-Switching Oligonucleotides
Source: Nucleic Acid Ther. 2021 Mar 25;31(2):172–81. doi: 10.1089/nat.2020.0907 (PMC7997716; doi:10.1089/nat.2020.0907)
Supplement: Supplemental data [file Supp_Fig5.docx]

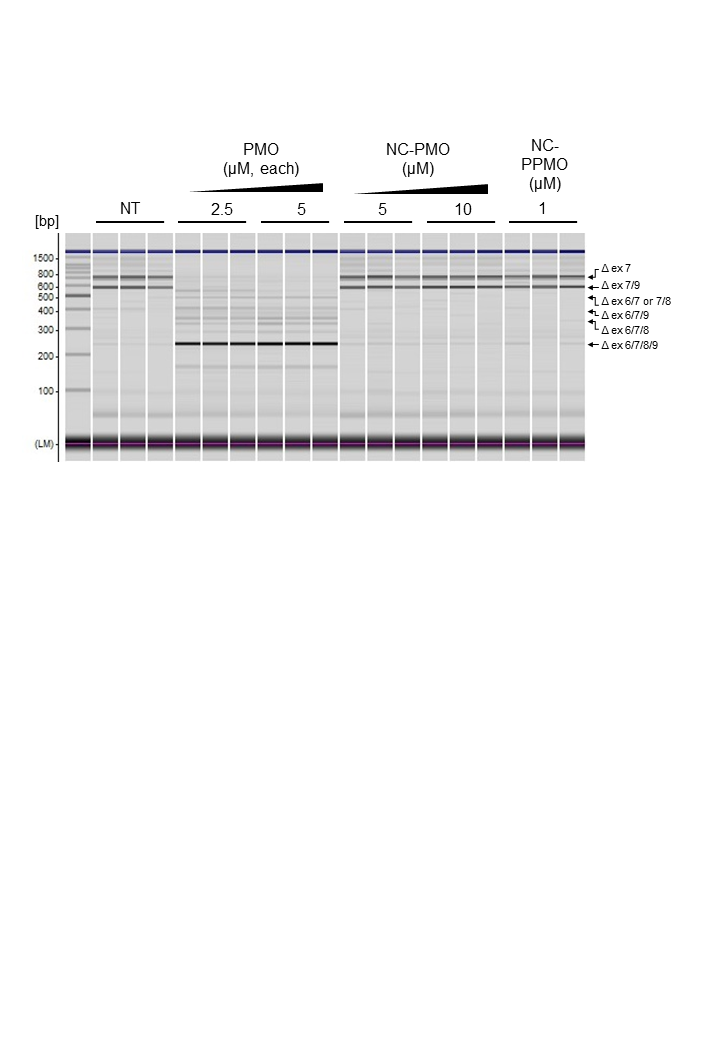


**Figure S5. Exon skipping analysis using negative control PMOs in immortalized myoblast line.** Image of RT-PCR analysis of *DMD* mRNA in immortalized myoblasts derived from CXMD_J_ dogs treated with PMOs for exon 6 and 8 skipping or standard control PMO (NC-PMO) and dog_C8A (invert antisense) conjugated to Pip8b2 (NC-PPMO) for negative control.
